# Patient-reported first-use usability of the noncommercial MyTARGET mobile application among adults with inflammatory bowel disease: a cross-sectional survey

**DOI:** 10.3389/fdgth.2026.1957062

**Published:** 2026-09-08

**Authors:** Daria Vasilyeva-Singer, Timo Rath, Christopher Kretzschmar, Johannes Knitza, Markus F. Neurath, Raja Atreya, Till Orlemann

**Affiliations:** 1 Department of Internal Medicine 1, Erlangen University Hospital, Friedrich-Alexander University of Erlangen-Nürnberg, Erlangen, Germany; 2 German Center for Immunotherapy, Friedrich-Alexander University Erlangen-Nürnberg and Erlangen University Hospital, Erlangen, Germany; 3 Department of Internal Medicine 3, Erlangen University Hospital, Friedrich-Alexander University of Erlangen-Nürnberg, Erlangen, Germany; 4 AGEIS, Université Grenoble Alpes, Grenoble, France; 5 Institute for Digital Medicine, University Hospitals of Giessen and Marburg, Philipps University of Marburg, Marburg, Germany

**Keywords:** crohn's disease, digital health, inflammatory bowel disease, mHealth, mobile application, telehealth usability, ulcerative colitis

## Abstract

**Background:**

Mobile health applications may support self-management in inflammatory bowel diseases (IBD), but evidence on first-use experiences with disease-specific mobile health applications is limited.

**Objective:**

To evaluate first-use usability of the noncommercial MyTARGET mobile application among adults with IBD in Germany and identify priorities for refinement.

**Methods:**

In this single-center cross-sectional usability survey, adults with Crohn's disease or ulcerative colitis installed MyTARGET on their own smartphones, received a 15-minute standardized introduction and independently explored the application for 45 min during one outpatient session. Participants completed a 13-item questionnaire adapted from the German Telehealth Usability Questionnaire on a 7-point Likert scale. Recommendation intention was assessed using the Net Promoter Score (NPS).

**Results:**

All 200 participants completed the questionnaire (mean age 39.1 years; SD, 14.0; 48.0% female); 127/200 (63.5%) had Crohn's disease and 73/200 (36.5%) had ulcerative colitis. Mean overall usability was 6.00 (SD, 0.57; 95% CI, 5.92–6.08). User-friendliness (mean, 6.65; SD, 0.61) and ease of learning (mean, 6.41; SD, 0.59) received the highest ratings, whereas error handling/troubleshooting (mean, 5.59; SD, 0.93) and perceived time savings were lower. The Net Promoter Score was +16 points. Overall usability correlated with recommendation score (Spearman *ρ* = 0.606; adjusted *p* < 0.001) and usefulness, interface quality and satisfaction/intention to use again showed stronger associations than user-friendliness.

**Conclusions:**

MyTARGET showed high patient-reported first-use usability in this selected outpatient sample. The findings identify aspects that could inform future refinement and suggest that ease of use alone may not determine willingness to recommend an IBD app.

## Introduction

Inflammatory bowel diseases (IBD), including Crohn's disease and ulcerative colitis, require lifelong disease monitoring, symptom assessment, medication management and regular communication between patients and healthcare professionals. Current IBD care increasingly follows a treat-to-target approach using both patient-reported outcomes and objective measures to guide therapeutic decisions (1–4).

Telemedicine is increasingly used in IBD care to support remote monitoring and communication between patients and healthcare professionals (5, 6). Mobile health applications may further support self-management by facilitating symptom documentation, medication management and access to disease-related information. Although such interventions are generally feasible, evidence on sustained use and long-term clinical benefit remains heterogeneous (6, 7). Patient-centered content is particularly important. Asadi et al. identified disease knowledge, medication, nutrition and lifestyle as key informational domains for an IBD self-management application (8), while participatory design work has highlighted symptom registration, knowledge resources, appointments and communication as patient-relevant functions (9). A recent systematic review found that IBD apps most commonly target education, monitoring, counselling and treatment support (10). Surveys and app-store evaluations nevertheless show substantial interest alongside limited real-world use and variable app quality (11, 12). Despite this growing literature, direct usability evidence for IBD-specific apps remains limited. Erlich et al. used patient and clinician user testing to identify modifications to the MyHealthyGut app involving tracking, ease of use and educational content (13), while Park et al. combined heuristic evaluation, four-week usability testing and focus groups to iteratively refine the WITH-Jang app (14). Previous studies have mainly examined app development or repeated use. Less is known about how patients perceive an IBD app during their first structured exposure in routine clinical care and which aspects of usability are related to willingness to recommend it. Difficulties with navigation, learnability or error recovery may influence acceptance from the beginning. MyTARGET is a noncommercial mobile application developed by the German IBD Competence Network in collaboration with the German Association of Office-Based Gastroenterologists specifically for patients with IBD (15). Its disease-specific functions include a symptom diary, a toilet finder, medication management and medication-related information, nutritional recommendations and information on ongoing IBD studies in Germany, together with additional organizational functions where available. This study evaluated patient-reported first-use usability of MyTARGET after a brief standardized introduction and one-time exploration in a routine outpatient setting. A secondary aim was to identify aspects of the user experience that could inform future refinement before longitudinal evaluation of engagement and clinical utility. By focusing on clinical onboarding, the study addresses a distinct stage of digital health adoption and may inform human-centered evaluation of disease-specific mHealth tools before longitudinal implementation.

## Methods

### Study design and reporting

We conducted a single-center, cross-sectional survey of patient-reported first-use usability of the MyTARGET mobile application among adults with inflammatory bowel disease. Participants completed the questionnaire immediately after completing the testing session. The study was designed as a formative evaluation of perceived initial usability before longitudinal assessment and was reported with reference to the STROBE statement for observational studies (16).

Adults treated at the IBD outpatient clinic of the University Hospital Erlangen were invited to participate between January and May 2025. Eligible participants were aged 18 years or older, had a physician-confirmed diagnosis of Crohn's disease or ulcerative colitis, were able to use a smartphone, had sufficient German-language proficiency and agreed to complete a one-time test of MyTARGET followed immediately by the questionnaire. Participation consisted of a single on-site testing session during the outpatient visit. Patients were excluded if they were unable to use a smartphone or had insufficient German-language proficiency. The number of eligible patients who declined participation and their reasons for nonparticipation were not systematically recorded. The findings therefore primarily reflect adults with IBD who were able and willing to use a smartphone application.

### MyTARGET mobile application and first-use testing procedure

During a routine outpatient visit, participants installed the version of the MyTARGET mobile application available for their smartphone operating system at the time of testing. The standardized 15-minute introduction provided an overview of the application's purpose, main navigation structure and principal functions and showed participants where the major self-management and organizational modules could be accessed. The introduction was intended as orientation to the application rather than step-by-step assistance or a fixed task sequence. Participants then explored the application independently during a 45-minute session, including symptom documentation, medication management, appointment functions, document storage and additional functions where available.

Questions arising during installation or initial exploration were answered pragmatically. This approach reflected routine clinical onboarding rather than a formal task-based usability protocol. Think-aloud procedures, structured task analyses or observational measures were not used. Accessibility was not formally assessed and testing was not conducted against ISO 9241 or another standards-based usability framework.

### Patient-reported usability

Perceived first-use usability was assessed using a 13-item questionnaire adapted from the German Telehealth Usability Questionnaire framework (17, 18). Items were rated on a 7-point Likert scale, with higher scores indicating more favorable evaluations. The complete item wording, response anchors, scoring and domain assignment are provided in Supplementary Material 1. The adapted items and their assignments were defined before analysis. Item-level results were considered primary, whereas domain scores were interpreted descriptively because some domains comprised only one or two items.

An overall usability score was calculated as the mean of all 13 TUQ-based items. Domain scores were calculated according to predefined item groupings: usefulness, ease of learning, user-friendliness, user interface quality, error handling and troubleshooting, acceptance and satisfaction and intention to use again. Because the questionnaire was adapted rather than administered in its original entirety, psychometric analyses were interpreted as measures of internal consistency in this sample and not as validation of a new instrument. Items concerning time savings or health care delivery were interpreted in the context of a self-management application rather than as a replacement for routine outpatient care.

### Recommendation intention

Recommendation intention was assessed using the 11-point NPS question, ranging from 0 to 10 (19). Respondents scoring 9 or 10 were classified as promoters, those scoring 7 or 8 as passives and those scoring 0 to 6 as detractors. NPS was calculated as the percentage of promoters minus the percentage of detractors and was interpreted separately from perceived usability.

### Statistical analysis

Analyses were descriptive and performed using IBM SPSS Statistics version 31 (IBM Corp., Armonk, NY, United States). Continuous variables are reported as mean and SD and, for primary descriptive estimates, with 95% CIs. CIs for means were calculated using a normal approximation. Categorical variables are reported as frequencies and percentages.

The 95% CI for the NPS was calculated using a large-sample multinomial approximation for the difference between the proportions of promoters and detractors. Internal consistency was assessed using Cronbach *α* for the overall scale and multi-item domains. Pearson interitem correlations are reported for 2-item domains in the main manuscript; Spearman-Brown coefficients are provided in Supplementary Material 2. Because the study was descriptive, no formal sample size calculation or confirmatory hypothesis testing was prespecified.

Post hoc exploratory analyses examined associations between recommendation intention and first-use usability. Spearman rank correlations were calculated between the continuous 0–10 recommendation score and the overall usability score and each usability domain. Overall and domain scores were also summarized across standard NPS categories (detractors, passives and promoters) and group differences were evaluated using Kruskal–Wallis tests. Epsilon-squared was calculated as an effect size for Kruskal–Wallis tests. To account for multiple comparisons, *p* values were adjusted within each family of eight analyses (overall score plus seven domains) using the Holm method. These analyses were exploratory and were not prespecified as confirmatory hypotheses. Exploratory analyses were performed using IBM SPSS Statistics version 31 (IBM Corp., Armonk, NY, United States).

### Ethics

The Ethics Committee of Friedrich-Alexander-Universität Erlangen-Nürnberg approved the study (No. 23-63-B). The study was conducted in accordance with institutional requirements and principles of good clinical practice. Before participation, all participants received a written patient information sheet describing the study, its purpose and its procedures and provided written informed consent.

## Results

### Participant characteristics

A total of 200 participants completed the questionnaire and were included in the analysis. There were no missing data for usability or recommendation items; all analyses were complete-case analyses. The mean age was 39.1 years (SD, 14.0) and 96/200 participants (48.0%) were female. Crohn's disease was reported by 127/200 participants (63.5%) and ulcerative colitis by 73/200 (36.5%). Mean disease duration was 13.1 years (SD, 9.9) and 146/200 participants (73.0%) reported a disease duration longer than 5 years. Almost all participants reported regular smartphone use (197/200, 98.5%), whereas 25/200 (12.5%) reported previous use of medical applications (Table 1).

**Table 1:** Patient characteristics (*N* = 200).

Characteristic	Value
Age (years), mean (SD)	39.1 (14.0)
Female, *n* (%)	96 (48.0)
Male, *n* (%)	104 (52.0)
Crohn's disease, *n* (%)	127 (63.5)
Ulcerative colitis, *n* (%)	73 (36.5)
Disease duration (years), mean (SD)	13.1 (9.9)
Disease duration > 5 years, *n* (%)	146 (73.0)
Smartphone use, *n* (%)	197 (98.5)
Tablet use, *n* (%)	108 (54.0)
Social media use, *n* (%)	139 (69.5)
Use of medical apps, *n* (%)	25 (12.5)

### First-use usability

Participants reported high first-use usability, with a mean overall score of 6.00 (SD, 0.57; 95% CI, 5.92–6.08) on the 7-point scale. The 13-item questionnaire showed good internal consistency in this sample (Cronbach *α* = 0.865). The highest domain scores were observed for user-friendliness (mean, 6.65; SD, 0.61), ease of learning (mean, 6.41; SD, 0.59) and user interface quality (mean, 6.18; SD, 0.65). Error handling and troubleshooting received the lowest domain score (mean, 5.59; SD, 0.93), although the mean remained above the scale midpoint (Table 2). Item-level results are provided in Supplementary Material 4. High ratings, defined as scores of 6 or 7, were most frequent for ease of learning and ease of use (195/200, 97.5% each). Perceived time savings (85/200, 42.5%) and helpfulness of error messages (107/200, 53.5%) received comparatively fewer high ratings.

**Table 2:** Patient-reported first-use usability scores and internal consistency. For 2-item domains, Pearson interitem correlations are reported; Spearman-Brown coefficients are provided in Supplementary Material 2.

Scale/Range (higher = better)	Number of items	Mean (SD)	Internal consistency
Overall first-use usability	13	6.00 (0.57); 95% CI 5.92–6.08	*α*=0.865
Usefulness	3	5.66 (0.90)	α=0.706
Ease of learning	2	6.41 (0.59)	r = 0.446
User-friendliness	1	6.65 (0.61)	Not applicable
User interface quality	2	6.18 (0.65)	r = 0.560
Error handling/troubleshooting	2	5.59 (0.93)	r = 0.573
Acceptance	1	5.79 (0.99)	Not applicable
Satisfaction/intention to use again	2	6.09 (0.81)	r = 0.666

### Recommendation intention

**Figure 1:**
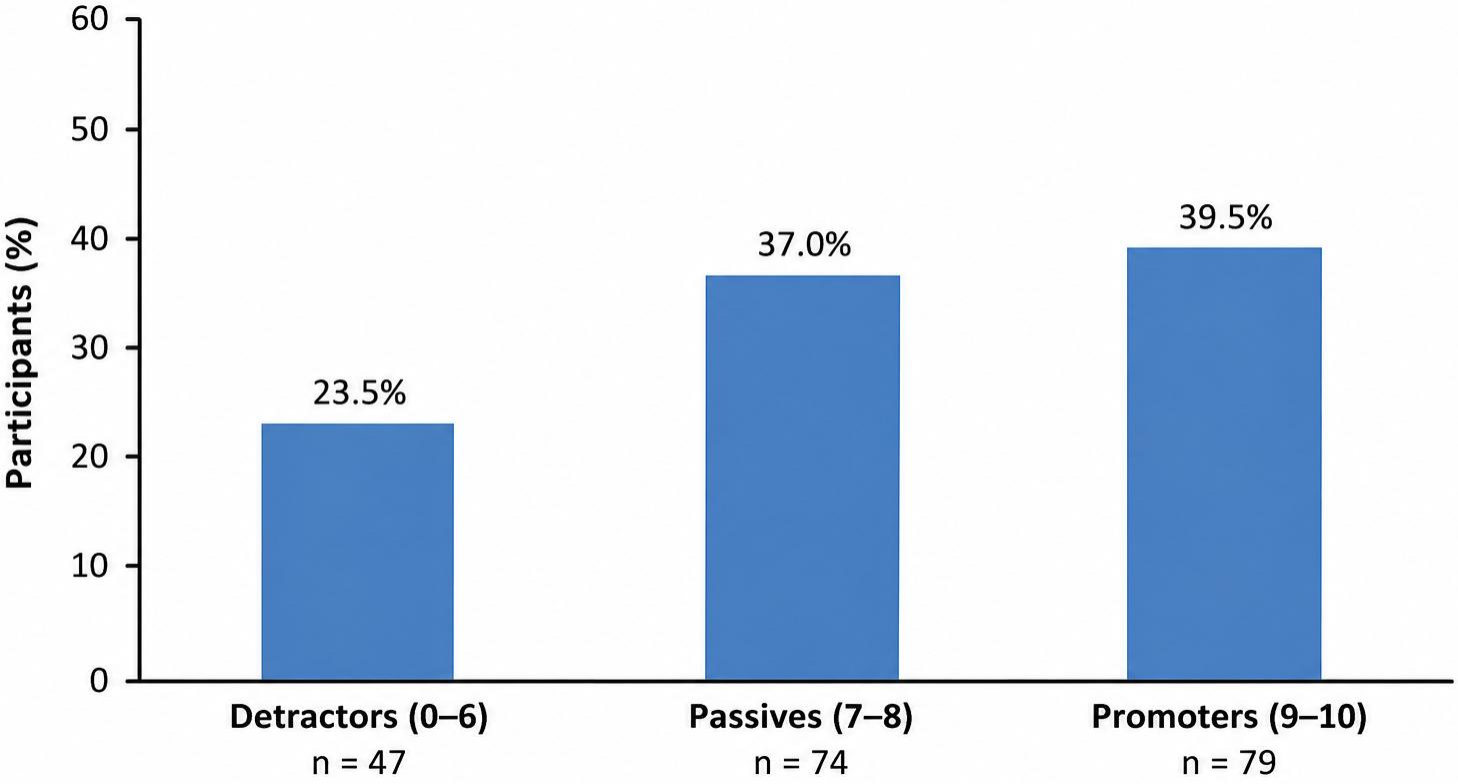
Recommendation intention according to Net promoter score (*N* = 200). The NPS was +16 points; 79/200 participants (39.5%) were promoters, 74/200 (37.0%) were passives and 47/200 (23.5%) were detractors.

The mean recommendation score was 7.59 (SD, 2.32; 95% CI, 7.27–7.91). According to standard NPS categories, 79/200 participants (39.5%) were promoters, 74/200 (37.0%) were classified as passives and 47/200 (23.5%) were detractors. The resulting NPS was +16 points (95% CI, +5.2 to +26.8; Figure 1).

### Exploratory associations with recommendation intention

Recommendation score was positively associated with overall first-use usability (Spearman *ρ* = 0.606; Holm-adjusted *p* < 0.001). Mean overall usability increased across NPS categories from 5.50 (SD, 0.59) among detractors to 5.91 (SD, 0.42) among passives and 6.37 (SD, 0.40) among promoters. The overall group difference was substantial (Kruskal–Wallis H = 69.59; Holm-adjusted *p* < 0.001; *ε*^2^ = 0.34).

At the domain level, the strongest correlations with recommendation score were observed for satisfaction/intention to use again (*ρ* = 0.625), usefulness (*ρ* = 0.528), user interface quality (*ρ* = 0.487) and ease of learning (*ρ* = 0.463). User-friendliness showed a weaker association (*ρ* = 0.182) despite receiving the highest overall rating. All eight overall/domain correlations remained statistically significant after Holm adjustment (Table 3). At item level, the strongest associations outside the satisfaction items were observed for perceived ability of the app to help with health concerns (*ρ* = 0.497), the ability to learn effective use quickly (*ρ* = 0.479), improved access to healthcare (*ρ* = 0.475) and enjoyment of the user interface (*ρ* = 0.458; Supplementary Material 4).

**Table 3:** Exploratory associations between first-use usability and recommendation intention.

Measure	Detractors (*n* = 47), mean (SD)	Passives (*n* = 74), mean (SD)	Promoters (*n* = 79), mean (SD)	Spearman *ρ*	Holm-adjusted p
Overall first-use usability	5.50 (0.59)	5.91 (0.42)	6.37 (0.40)	0.606	<0.001
Usefulness	5.00 (1.02)	5.53 (0.66)	6.18 (0.70)	0.528	<0.001
Ease of learning	5.97 (0.66)	6.41 (0.53)	6.68 (0.42)	0.463	<0.001
User-friendliness	6.49 (0.62)	6.66 (0.50)	6.73 (0.67)	0.182	0.010
User interface quality	5.76 (0.72)	6.09 (0.58)	6.53 (0.48)	0.487	<0.001
Error handling/troubleshooting	5.40 (0.91)	5.35 (0.94)	5.94 (0.84)	0.279	<0.001
Acceptance	5.28 (1.14)	5.73 (0.82)	6.15 (0.89)	0.350	<0.001
Satisfaction/intention to use again	5.24 (0.90)	6.09 (0.58)	6.58 (0.46)	0.625	<0.001

NPS categories: detractors=0–6, passives=7–8, promoters=9–10. Spearman correlations use the continuous 0–10 recommendation score. *P* values are Holm-adjusted across the eight overall/domain correlations.

## Discussion

### Principal findings

In this cross-sectional survey of 200 adults with inflammatory bowel disease, participants reported high first-use usability of the noncommercial MyTARGET mobile application following a standardized introduction and one-time exploration. Ease of learning and user-friendliness received the highest ratings, whereas error handling, troubleshooting and perceived time savings were comparatively lower. *Post hoc* exploratory analyses showed that overall usability was substantially higher among promoters than detractors and was moderately to strongly associated with the continuous recommendation score. Satisfaction/intention to use again and usefulness showed the strongest domain-level associations with recommendation intention, while user-friendliness remained uniformly high and showed only a weak association.

These findings suggest that ease of operation alone does not fully explain willingness to recommend a disease-specific mHealth application. Perceived usefulness, satisfaction, interface experience and learnability may add distinct value beyond basic ease of use. The strong association for satisfaction/intention to use again is partly expected because this construct is conceptually close to recommendation intention. Because these analyses were *post hoc* and cross-sectional, they should be interpreted as hypothesis-generating associations rather than causal determinants of recommendation behavior.

### Comparison with previous research

The findings complement broader evidence that digital health can support IBD self-management while long-term engagement and effectiveness vary across interventions (6, 7). Direct IBD app studies provide particularly relevant context. Erlich et al. used patient and clinician user testing to identify modifications to MyHealthyGut related to tracking, ease of use and educational content (13). Park et al. combined expert heuristic evaluation, four-week patient usability testing and focus groups to refine WITH-Jang (14). Zhen et al. found the MyGut platform feasible and acceptable in prospective IBD care but also highlighted the challenge of sustained use (20). Digital IBD interventions continue to diversify; iACT4IBD, for example, evaluated a brief online psychological intervention with clinical outcomes rather than usability (21). Together, these studies span design, usability, feasibility and clinical-effectiveness stages, whereas the present study focuses specifically on the immediate onboarding stage.

Disease-specific content may also shape perceived value. Asadi et al. identified medication, nutrition, disease information and lifestyle as important informational needs for IBD self-management applications (8) and participatory design work has similarly emphasized symptom registration, knowledge resources, appointments and communication as patient-relevant functions (9). Recent qualitative work on medication literacy highlights the complexity of medication-information practices among people with IBD (22). MyTARGET's symptom diary, medication information, nutrition recommendations, toilet finder and study information address several such disease-specific needs. Comparable evaluations outside IBD also suggest that strong technical usability does not necessarily translate directly into subjective value. A participatory-design mobile application for diabetic retinopathy received high patient-reported usability (23), whereas a recent MARS evaluation of fatty liver self-care applications found functionality to be the strongest dimension and subjective quality the weakest (24). Together with our exploratory findings, these studies support evaluating multiple dimensions of user experience rather than relying on a single global acceptability measure.

### Implications for digital health design

Because MyTARGET is a third-party application and the authors had no role in its development or governance, these findings should be understood as an independent external evaluation. They identify aspects of the user experience that can be communicated to the app developers and may also inform evaluation of similar disease-specific digital health tools. High ratings for learnability and user-friendliness suggest that brief outpatient onboarding can be feasible, whereas lower ratings for error handling and perceived time savings indicate the importance of clear error messages, recovery options and efficient workflows.

More broadly, first-use evaluations may benefit from separating learnability and user-friendliness from usefulness, satisfaction, error recovery and recommendation intention. In the present study, globally high ease-of-use ratings coexisted with substantially lower recommendation intention among some participants, suggesting that successful onboarding does not guarantee sufficient perceived value to recommend the application. Disease-specific design should therefore be considered together with functional relevance to the intended user population. Task-based usability testing could further identify where errors occur and whether users can recover without assistance (25). These findings should guide formative evaluation rather than be interpreted as evidence of implementation readiness or clinical effectiveness.

### Strengths and limitations

Strengths include the sample of 200 adults with IBD, complete data for the primary outcomes and assessment immediately after initial interaction with a disease-specific self-management application. The questionnaire was based on the validated German TUQ framework and showed good internal consistency in this sample. The study also addresses a distinct stage in digital health adoption that is not captured by surveys of expectations or by evaluations conducted only after prolonged use.

Several limitations should be considered. First, this was a single-center, cross-sectional survey after a brief standardized introduction and one-time exploration. It measured perceived first-use usability rather than prolonged real-world use. Second, outcomes were self-reported and objective measures such as task completion, completion time, navigation errors, or observational usability data were not collected. Third, participants were recruited from a tertiary IBD outpatient clinic and eligibility required the ability to use a smartphone. Almost all participants were regular smartphone users, limiting generalizability to people with less digital experience or access. Fourth, nonparticipation was not systematically documented, so selection bias cannot be quantified. Fifth, the adapted 13-item questionnaire did not represent the complete original TUQ and the internal consistency analyses do not constitute validation of a new instrument. Single-item and 2-item domains should therefore be interpreted cautiously. The high proportion of ratings at the upper end of the scale for several items suggests possible ceiling effects, which may have limited discrimination among favorable user experiences. Sixth, accessibility was not formally assessed and the test was not conducted against ISO 9241 or another standards-based usability framework. Qualitative explanations for ratings and engagement/log metrics were also not collected. Finally, the *post hoc* exploratory analyses were cross-sectional and cannot establish causal determinants of recommendation behavior. NPS should therefore be interpreted as a complementary measure of willingness to recommend the application and not as evidence of long-term acceptance, sustained use or clinical effectiveness.

### Future research

Future studies should evaluate sustained engagement with the MyTARGET application under routine clinical conditions, including frequency and duration of use, feature-specific engagement and reasons for discontinuation. Combining patient-reported usability with task-based observation, standards-based accessibility assessment, application log data and qualitative interviews would provide a more complete account of how and why patients engage with disease-specific digital tools (25, 26). Longitudinal studies are also needed to determine whether favorable first-use usability translates into continued use, improved self-management, or clinically meaningful outcomes. Individual differences in technology-related attitudes or personality may additionally help explain heterogeneity in adoption and recommendation intention.

## Conclusions

Adults with inflammatory bowel disease in this selected outpatient sample reported high first-use usability of the noncommercial MyTARGET mobile application after initial exposure. Ease of learning and user-friendliness were rated particularly favorable, whereas error handling, troubleshooting and perceived time savings emerged as priorities for refinement. Exploratory analyses further suggested that recommendation intention was more strongly associated with perceived usefulness, satisfaction and interface quality than with user-friendliness alone. These findings provide formative, externally generated evidence for human-centered evaluation of disease-specific mHealth applications and a basis for longitudinal assessment of sustained engagement, routine use and clinical utility.

## Data Availability

The anonymized aggregated data supporting the conclusions of this article are available from the corresponding author upon justified request, subject to institutional and ethical requirements.
