# Supplementary material for: Patient-reported first-use usability of the noncommercial MyTARGET mobile application among adults with inflammatory bowel disease: a cross-sectional survey
Source: Front Digit Health. 2026 Sep 8;8:1957062. doi: 10.3389/fdgth.2026.1957062 (PMC13593808; doi:10.3389/fdgth.2026.1957062)
Supplement: Supplementary file 1 [file Table1.docx]

**Supplementary Material 1. TUQ-based questionnaire items, response anchors, scoring and domain assignment**

Response anchors: 1=strongly disagree to 7=strongly agree

| No. | Item | Domain |
| --- | --- | --- |
| 1 | The app improves my access to healthcare | Usefulness |
| 2 | The app saves me a trip to the hospital or my doctor’s office | Usefulness |
| 3 | The app can help me with my health concerns | Usefulness |
| 4 | The app was easy to learn how to use | Ease of learning |
| 5 | The app is easy to use | User-friendliness |
| 6 | I think I could quickly learn to use the app effectively | Ease of learning |
| 7 | The app’s user interface is attractively designed | User interface quality |
| 8 | I enjoy using the app’s user interface | User interface quality |
| 9 | Whenever I encountered an error while using the app, I was able to resolve it quickly and easily | Error handling/troubleshooting |
| 10 | The app’s error messages are clear and helpful for troubleshooting | Error handling/troubleshooting |
| 11 | It is acceptable to receive healthcare through the app | Acceptance |
| 12 | I would use the app again | Satisfaction/intention to use again |
| 13 | Overall, I am satisfied with the app | Satisfaction/intention to use again |

Scoring: Higher scores indicate more favorable usability evaluation. The overall usability score was calculated as the mean of all 13 items. Domain scores were calculated as the mean of the items assigned to each domain.
