# Supplementary material for: Patient-reported first-use usability of the noncommercial MyTARGET mobile application among adults with inflammatory bowel disease: a cross-sectional survey
Source: Front Digit Health. 2026 Sep 8;8:1957062. doi: 10.3389/fdgth.2026.1957062 (PMC13593808; doi:10.3389/fdgth.2026.1957062)
Supplement: Supplementary file 2 [file Table2.docx]

**Supplementary Material 2.**

**Interitem reliability for domains with 2 items**

Spearman-Brown coefficient = 2r / (1 + r).

| **Domain (2 items)** | **Pearson r** | **Spearman-Brown coefficient** |
| --- | --- | --- |
| Ease of learning | 0.446 | 0.616 |
| User interface quality | 0.560 | 0.718 |
| Error handling/troubleshooting | 0.573 | 0.729 |
| Satisfaction/intention to use again | 0.666 | 0.800 |
