# Supplementary material for: Patient-reported first-use usability of the noncommercial MyTARGET mobile application among adults with inflammatory bowel disease: a cross-sectional survey
Source: Front Digit Health. 2026 Sep 8;8:1957062. doi: 10.3389/fdgth.2026.1957062 (PMC13593808; doi:10.3389/fdgth.2026.1957062)
Supplement: Supplementary file 3 [file Table3.docx]

**Supplementary Material 3.**

**STROBE Checklist:** Checklist for cross-sectional observational studies.

| **Item** | **Recommendation** | **Where addressed / notes** |
| --- | --- | --- |
| Title/Abstract | Indicate study design and provide informative summary. | Title; Abstract |
| Introduction - Background | Explain scientific background and rationale. | Introduction |
| Introduction - Objectives | State specific objectives. | End of Introduction |
| Methods - Study design | Present key elements of study design early. | Methods: Study design and reporting |
| Methods - Setting | Describe setting, locations, and relevant dates. | Methods: Study design and reporting; recruitment from January to May 2025 |
| Methods - Participants | Give eligibility criteria and participant selection. | Methods: Study design and reporting |
| Methods - Variables | Define outcomes and variables. | Methods: Patient-reported usability; Recommendation intention |
| Methods - Data sources/measurement | Give sources of data and details of assessment methods. | Methods: MyTARGET mobile application and first-use testing procedure; Patient-reported usability; Recommendation intention. |
| Methods - Bias | Describe efforts to address potential sources of bias. | Methods: standardized introduction and exploration procedure; Discussion: Strengths and limitations. |
| Methods - Study size | Explain how study size was arrived at. | Methods: Statistical analysis; no formal sample size calculation was performed |
| Methods - Quantitative variables | Explain handling of quantitative variables. | Methods: Statistical analysis |
| Methods - Statistical methods | Describe statistical methods and handling of missing data. | Methods: Statistical analysis; descriptive analyses, confidence intervals, internal consistency, post hoc exploratory Spearman correlations, NPS-category comparisons, Holm adjustment and handling of complete cases are described. |
| Results - Participants | Report numbers at each stage. | Results: Participant characteristics; approached and nonparticipation counts were not available |
| Results - Descriptive data | Report participant characteristics. | Results: Participant characteristics; Table 1 |
| Results - Outcome data | Report outcome data. | Results: First-use usability; Recommendation intention; Exploratory associations with recommendation intention; Table 2; Table 3; Figure 1; Supplementary Material 4. |
| Results - Main results | Give estimates and precision. | Results: First-use usability; Recommendation intention; Exploratory associations with recommendation intention; 95% CIs and exploratory effect estimates are reported. |
| Results - Other analyses | Report other analyses. | Results: Exploratory associations with recommendation intention; Table 3; Supplementary Material 4. These analyses are explicitly identified as post hoc and exploratory. |
| Discussion - Key results | Summarize key results. | Discussion: Principal findings |
| Discussion - Limitations | Discuss limitations. | Discussion: Strengths and limitations, including single-center design, subjective outcomes, adapted TUQ, ceiling effects, absence of formal accessibility/ISO testing, qualitative/log metrics and the post hoc nature of exploratory analyses. |
| Discussion - Interpretation | Provide cautious interpretation. | Discussion: Principal findings; Comparison with previous research; Implications for digital health design; Conclusions. Post hoc associations are interpreted as hypothesis-generating rather than causal. |
| Discussion - Generalizability | Discuss external validity. | Discussion: Strengths and limitations |
| Other information - Funding | Give source of funding and role of funders. | Funding |
