# Supplementary material for: Patient-reported first-use usability of the noncommercial MyTARGET mobile application among adults with inflammatory bowel disease: a cross-sectional survey
Source: Front Digit Health. 2026 Sep 8;8:1957062. doi: 10.3389/fdgth.2026.1957062 (PMC13593808; doi:10.3389/fdgth.2026.1957062)
Supplement: Supplementary file 4 [file Table4.docx]

**Supplementary Material 4.**

**Item-level usability results for the TUQ-based questionnaire and exploratory associations with recommendation intention**

High ratings were defined as scores of 6 or 7 on the 7-point scale. High scores indicate more favorable usability evaluation. Exploratory Spearman rank correlations use the continuous 0–10 recommendation score.

| **No.** | **Item** | **Domain** | **Mean (SD)** | **Median (IQR)** | **Scores 6–7, n (%)** | **Spearman ρ with recommendation score** | **Holm-adjusted p** |
| --- | --- | --- | --- | --- | --- | --- | --- |
| 1 | The app improves my access to healthcare | Usefulness | 5.92 (0.99) | 6 (5–7) | 143 (71.5) | 0.475 | <0.001 |
| 2 | The app saves me a trip to the hospital or my doctor’s office | Usefulness | 5.01 (1.48) | 5 (4–6) | 85 (42.5) | 0.380 | <0.001 |
| 3 | The app can help me with my health concerns | Usefulness | 6.07 (0.82) | 6 (6–7) | 158 (79.0) | 0.497 | <0.001 |
| 4 | The app was easy to learn how to use | Ease of learning | 6.66 (0.53) | 7 (6–7) | 195 (97.5) | 0.235 | 0.002 |
| 5 | The app is easy to use | User-friendliness | 6.65 (0.61) | 7 (6–7) | 195 (97.5) | 0.182 | 0.010 |
| 6 | I think I could quickly learn to use the app effectively | Ease of learning | 6.17 (0.84) | 6 (6–7) | 166 (83.0) | 0.479 | <0.001 |
| 7 | The app’s user interface is attractively designed | User interface quality | 6.41 (0.70) | 7 (6–7) | 183 (91.5) | 0.412 | <0.001 |
| 8 | I enjoy using the app’s user interface | User interface quality | 5.96 (0.79) | 6 (6–6) | 152 (76.0) | 0.458 | <0.001 |
| 9 | Whenever I encountered an error while using the app, I was able to resolve it quickly and easily | Error handling/troubleshooting | 5.81 (1.00) | 6 (5–6) | 147 (73.5) | 0.220 | 0.003 |
| 10 | The app’s error messages are clear and helpful for troubleshooting | Error handling/troubleshooting | 5.38 (1.10) | 6 (4–6) | 107 (53.5) | 0.275 | <0.001 |
| 11 | It is acceptable to receive healthcare through the app | Acceptance | 5.79 (0.99) | 6 (5–6) | 134 (67.0) | 0.350 | <0.001 |
| 12 | I would use the app again | Satisfaction/intention to use again | 6.06 (0.99) | 6 (5.8–7) | 150 (75.0) | 0.586 | <0.001 |
| 13 | Overall, I am satisfied with the app | Satisfaction/intention to use again | 6.11 (0.78) | 6 (6–7) | 169 (84.5) | 0.553 | <0.001 |

Note: Fractional quartile values may occur when percentile interpolation is used. Correlation analyses were post hoc and exploratory; p values were Holm-adjusted across 13 item-level correlations.
